# Supplementary material for: Is large improvement in efficiency of impulsive noise removal in color images still possible?
Source: PLoS One. 2021 Jun 28;16(6):e0253117. doi: 10.1371/journal.pone.0253117 (PMC8238199; doi:10.1371/journal.pone.0253117)
Supplement: S1 File — (PDF) [file pone.0253117.s001.pdf]

Additional examples

Lukasz Malinski, Krystian Radlak, Bogdan Smolka

1 Remarks

In order to provide the readers with more comprehensive comparison of the obtained results, additional corrupted test images: CRAYONS, GIRL, HAND, RAFTING, PEACOCK and CAPS, have been selected. For those images both visual results of final impulse suppression performance and aim-plots showing the impulse detection efficiency have been presented. Also for better coverage of all obtained results, image HAND is presented for $\rho = 0.1$, PEACOCK for $\rho = 0.5$ and CAPS using the ACWD algorithm, which provided the lowest detection efficiency.

The following additional remarks can be drawn:

- All impulsive noise suppression algorithms perform very well in homogeneous image regions (Fig. 1).
- All techniques except CNNE introduce more or less damage to edges within the image (Figs. 1 and 3).
- Originally occurring small details are well-preserved if CNND is used (Fig. 5).
- Sometimes impulses are so similar to the surroundings, that they are omitted even by very good detectors. Those masked impulses are not removed by any suppression algorithm (Fig. 7).
- Even the best combination of the detector and estimator - CNND-CNNE encounter problem with removing all explicit impulses for high contamination density (Fig. 9 with $\rho = 0.5$).
- Some less efficient algorithms introduce lots of visible artifacts in textured image regions for strong enough noise (Fig. 9).
- For images with homogeneous regions like CAPS, even using the least efficient detector provides acceptable results. However, some impulse detection techniques introduce visible damage to more detailed regions (Fig. 11).
- For strong impulsive noise, the majority of reduction imperfections are cased by TP results (Fig. 10), even if the least efficient detector is used. This can be explained by the strong impact of the neighboring impulses in the surroundings of restored pixels.

2 Figures

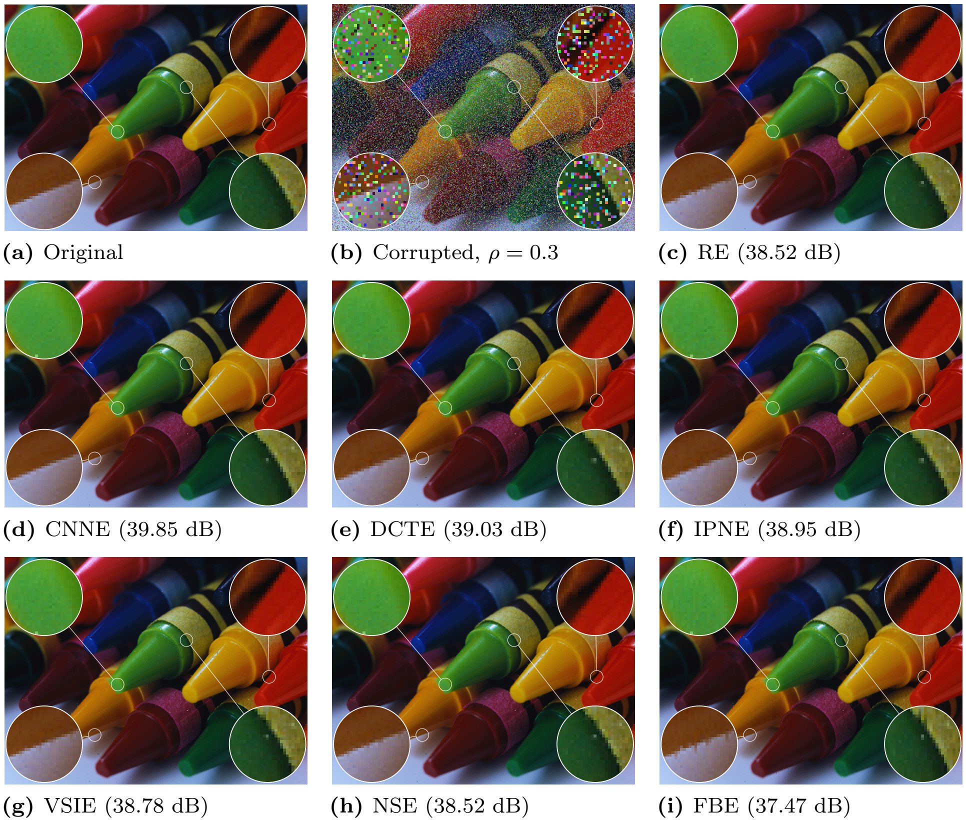

(a) Original (b) Corrupted, $\rho = 0.3$ (c) RE (38.52 dB)
(d) CNNE (39.85 dB) (e) DCTE (39.03 dB) (f) IPNE (38.95 dB)
(g) VSIE (38.78 dB) (h) NSE (38.52 dB) (i) FBE (37.47 dB)

Fig 1. Impulse suppression performance on CRAYONS image ($\rho = 0.3$) using CNND.

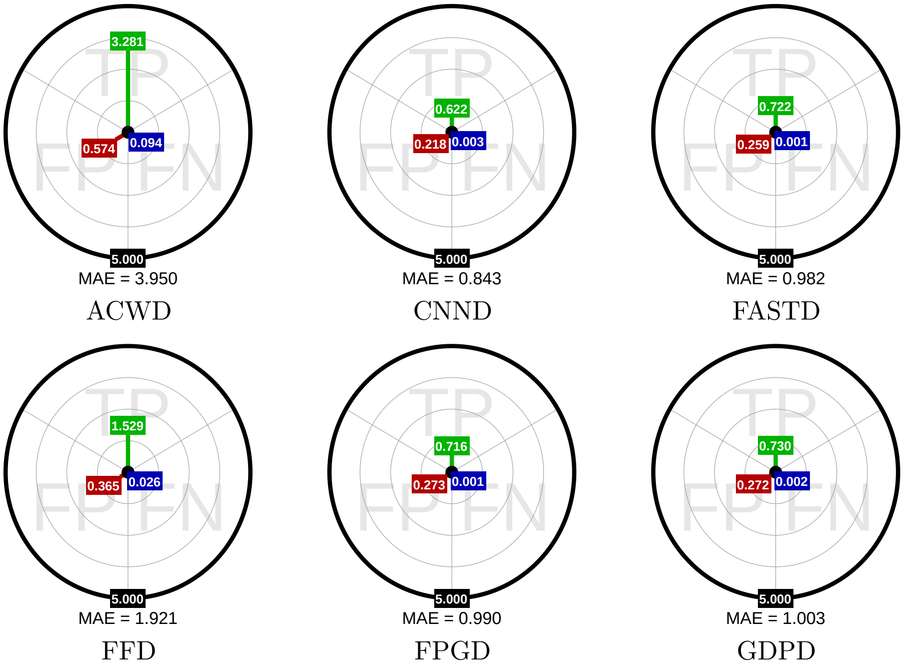

Fig 2. Aim-plots for CRAYONS image, $\rho = 0.3$ and CNNE.

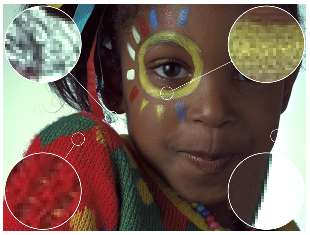
(a) Original

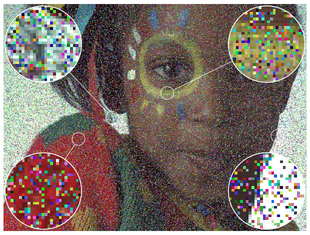
(b) Corrupted, $\rho = 0.3$

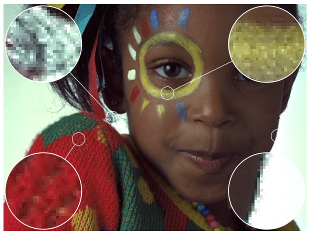
(c) RE (34.67 dB)

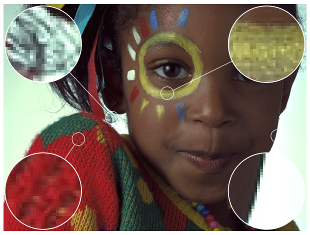
(d) CNNE (37.33 dB)

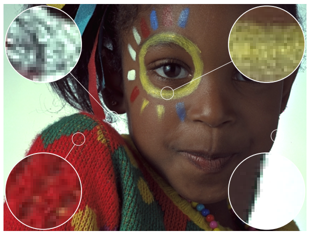
(e) DCTE (35.93 dB)

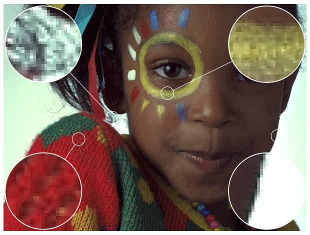
(f) IPNE (35.97 dB)

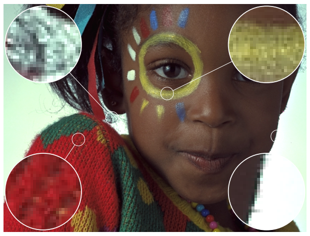
(g) VSIE (35.27 dB)

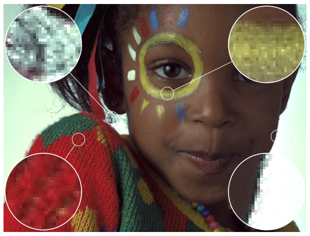
(h) NSE (34.38 dB)

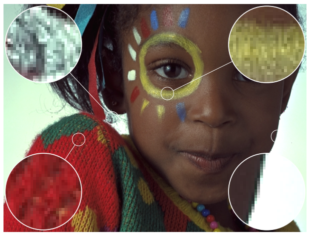
(i) FBE (34.48 dB)

Fig 3. Impulse suppression performance on GIRL image ($\rho = 0.3$) using CNND.

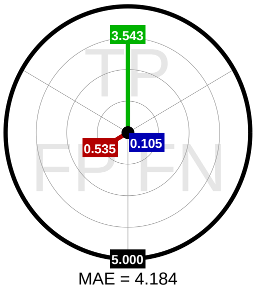

ACWD

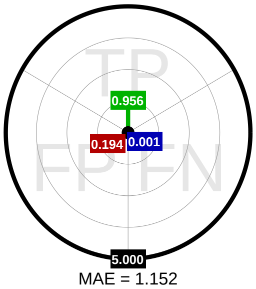

CNND

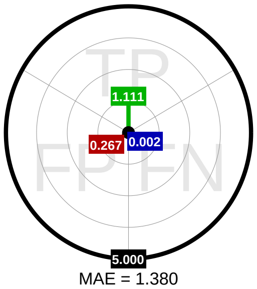

FASTD

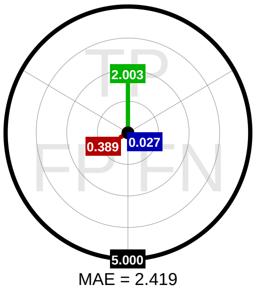

FFD

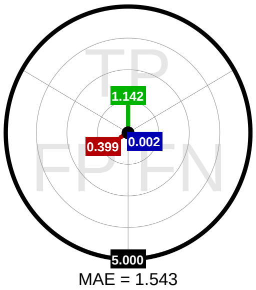

FPGD

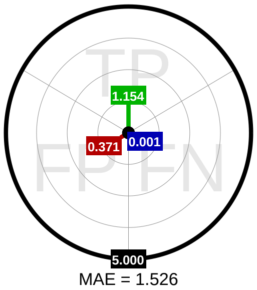

GDPD

Fig 4. Aim-plots for GIRL image, $\rho = 0.3$ and CNNE.

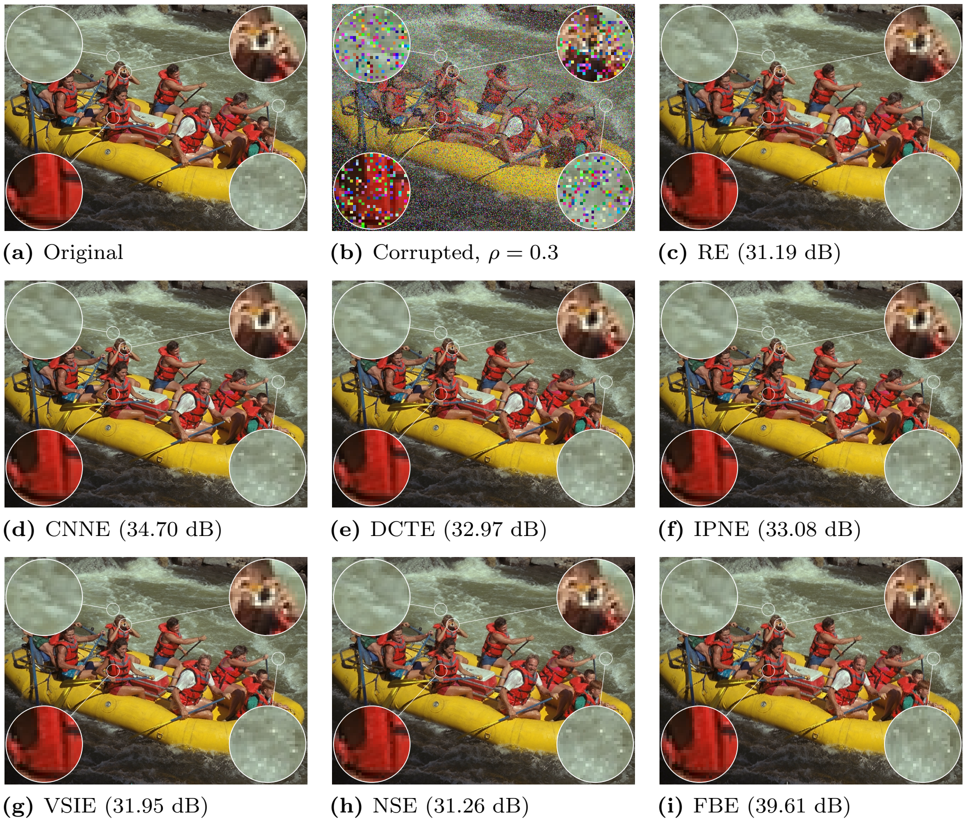

(a) Original (b) Corrupted, $\rho = 0.3$ (c) RE (31.19 dB)
(d) CNNE (34.70 dB) (e) DCTE (32.97 dB) (f) IPNE (33.08 dB)
(g) VSIE (31.95 dB) (h) NSE (31.26 dB) (i) FBE (39.61 dB)

Fig 5. Impulse suppression performance on RAFTING image ($\rho = 0.3$) using CNND.

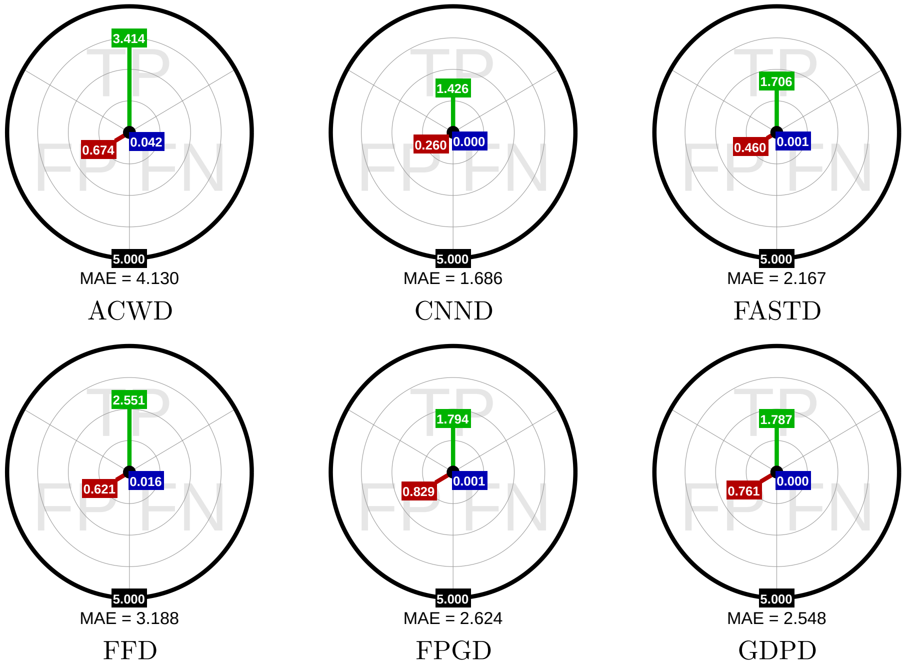

Fig 6. Aim-plots for RAFTING image, $\rho = 0.3$ and CNNE.

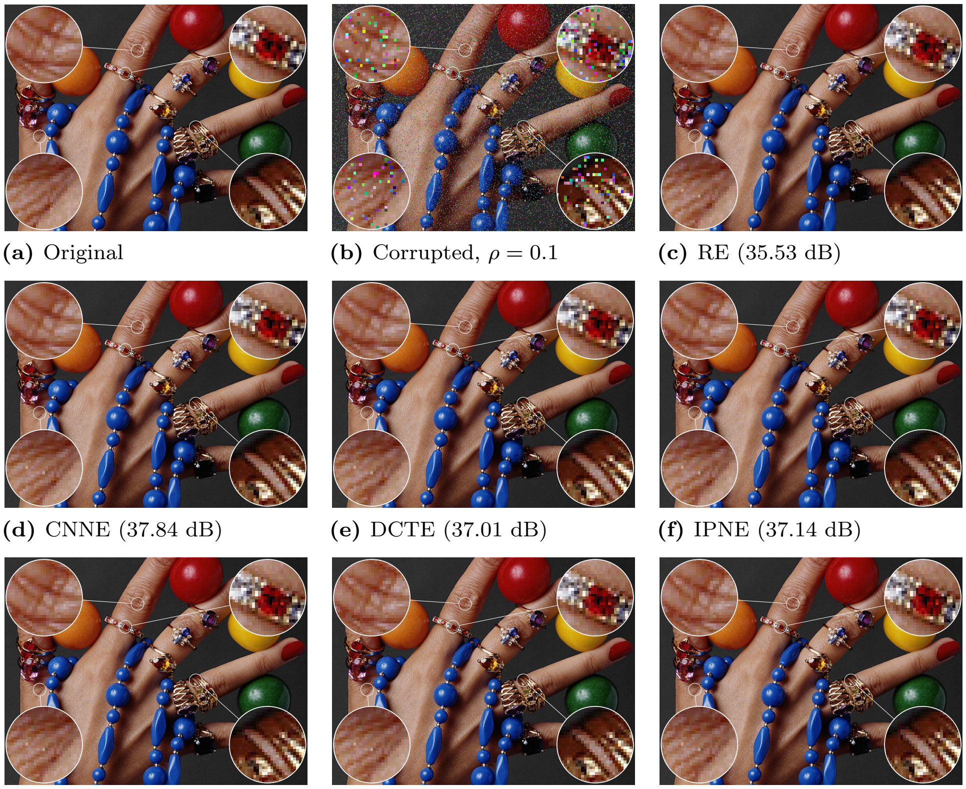

(a) Original (b) Corrupted, $\rho = 0.1$ (c) RE (35.53 dB)

(d) CNNE (37.84 dB) (e) DCTE (37.01 dB) (f) IPNE (37.14 dB)

(g) VSIE (36.42 dB) (h) NSE (35.49 dB) (i) FBE (35.03 dB)

Fig 7. Impulse suppression performance on HAND image ($\rho = 0.1$) using CNND.

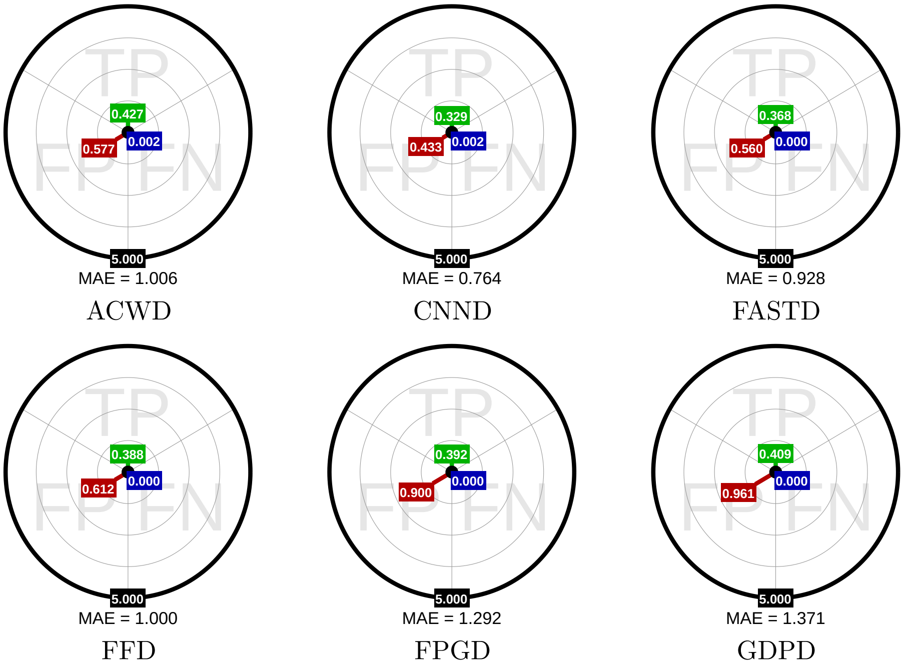

Fig 8. Aim-plots for HAND image, $\rho = 0.1$ and CNNE.

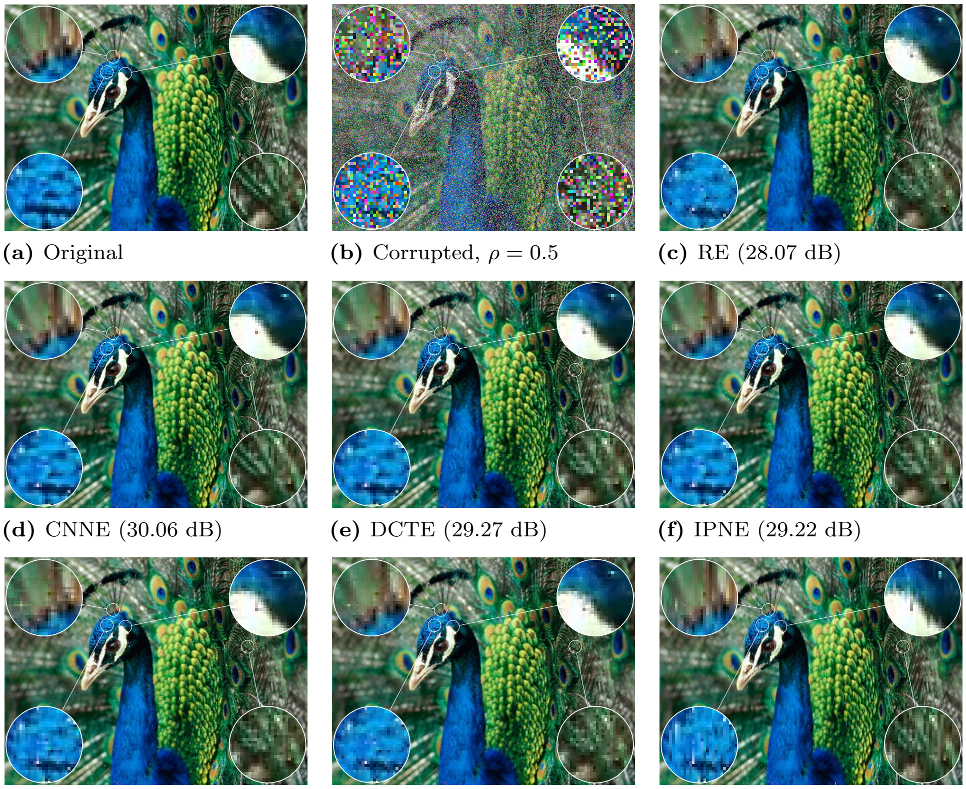

(a) Original **(b)** Corrupted, $\rho = 0.5$ **(c)** RE (28.07 dB)

(d) CNNE (30.06 dB) **(e)** DCTE (29.27 dB) **(f)** IPNE (29.22 dB)

(g) VSIE (28.15 dB) **(h)** NSE (28.12 dB) **(i)** FBE (27.30 dB)

Fig 9. Impulse suppression performance on PEACOCK image ($\rho = 0.5$) using CNND.

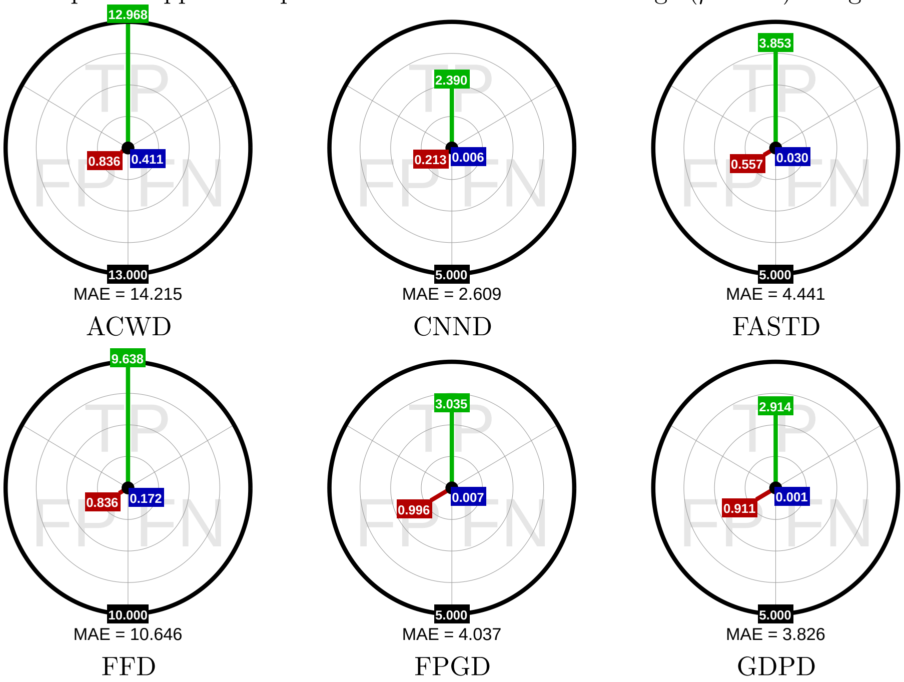

Fig 10. Aim-plots for PEACOCK image, $\rho = 0.5$ and CNNE.

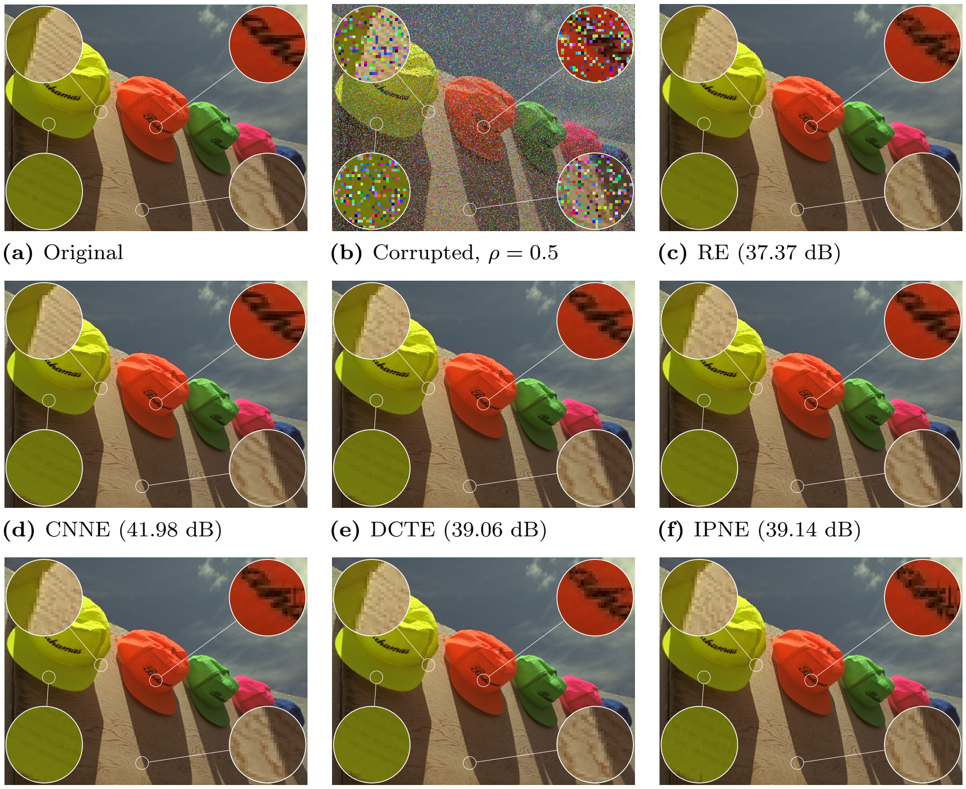

(a) Original (b) Corrupted, $\rho = 0.5$ (c) RE (37.37 dB)

(d) CNNE (41.98 dB) (e) DCTE (39.06 dB) (f) IPNE (39.14 dB)

(g) VSIE (38.20 dB) (h) NSE (37.22 dB) (i) FBE (35.52 dB)

Fig 11. Impulse suppression performance on CAPS image ($\rho = 0.3$) using ACWD.

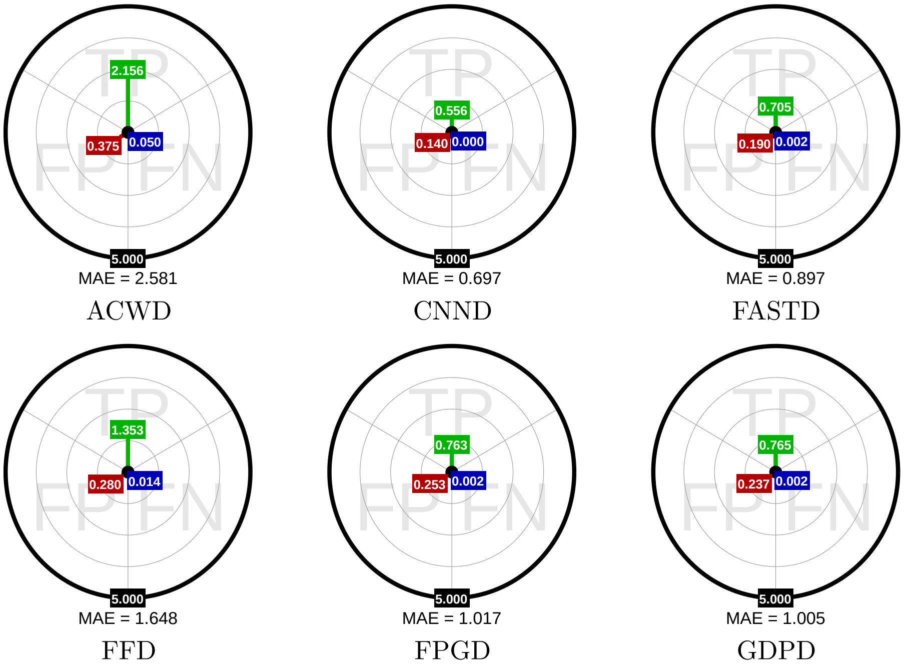

Fig 12. Aim-plots for CAPS image, $\rho = 0.3$ and CNNE.
