# Supplementary material for: Is large improvement in efficiency of impulsive noise removal in color images still possible?
Source: PLoS One. 2021 Jun 28;16(6):e0253117. doi: 10.1371/journal.pone.0253117 (PMC8238199; doi:10.1371/journal.pone.0253117)
Supplement: Attachment — Submitted filename: Response to Reviewers.pdf [file pone.0253117.s002.pdf]

Response to the Reviewers Comments

Lukasz Malinski, Krystian Radlak, Bogdan Smolka

Dear Editor and Reviewers,
we are very grateful for providing us with insightful review. The comments are very valuable for us and we did our best to improve the quality of our manuscript. In the following pages we provide our point-by-point responses to each of the comments.

Editor:

> Please ensure that your manuscript meets PLOS ONE's style requirements, including those for file naming.

We have revised our paper to better meet PLOS ONE's style requirements.

> In your Data Availability statement, it is unclear why you have selected 'No - some restrictions will apply'. PLOS defines a study's minimal data set as the underlying data used to reach the conclusions drawn in the manuscript and any additional data required to replicate the reported study findings in their entirety. All PLOS journals require that the minimal data set be made fully available. For more information about our data policy, please see http://journals.plos.org/plosone/s/data-availability.

We are sorry that the information we provided was not correct. In the revised version, we provided a link to the entire 7 GB dataset (stored on KAGGLE) containing the original, corrupted and restored images using every combination of the considered algorithms in the paper. In this way the readers can verify the results and also use them in their research.

> We note that you have provided funding information that is not currently declared in your Funding Statement. However, funding information should not appear in the Acknowledgments section or other areas of your manuscript. We will only publish funding information present in the Funding Statement section of the online submission form.
>
> Please remove any funding-related text from the manuscript and let us know how you would like to update your Funding Statement. Currently, your Funding Statement reads as follows:
>
> "The funders had no role in study design, data collection and analysis, decision to publish, or preparation of the manuscript."
>
> Please include your amended statements within your cover letter; we will change the online submission form on your behalf.

We have removed the founding statements from the manuscript and updated our cover letter as instructed

Reviewer 1:

> Overall, the article is well organized and its presentation is good. However, it also has some weaknesses. First, the style of subtitles in this article is not very clear. Primary and secondary subtitles are distinguished by font size, which is easy to misread.

We are using the available PLOS ONE LaTeX style, which defines certain section and subsections appearance, and we do not dare to change the official style. Therefore we have no influence on the appearance of subtitles in the preprint version. Hopefully, the paper's final appearance will be improved in the final version of the article, prepared by typesetters.

Reviewer 1:

> Second, the experiment is only implemented on the synthetic dataset. If some visual comparisons can be made on the real data, the analysis will be more persuasive.distinguished by font size, which is easy to misread.

Reviewer 2:

> The experiments have been performed using dataset containing 100 images with 640x480 pixels resolution. In the experiments, random noise was introduced and then denoising methods were employed. It will be better if authors can include results for real world noisy images where the noise is inherent due to camera/microscope limitations. A figure (Like Fig. 5) showing denoised images using various detection/estimation for a noisy image will improve the results.

Thank you for this suggestion. Experiments on real data have been added. Two images naturally contaminated by impulsive noise have been restored using the best combination of detector-estimator and presented in the paper. We provided an example of denoising the cDNA image and an old work of art, which we digitized from a photographic plate using a high quality scanner.

Reviewer 1:

> Last but not least, the details of the experiment are not clear enough. For example, the dataset and some hyper parameters are not clearly introduced in the training of CNN.

Table 2 summarizing the training parameters of the used CNN has been added to the paper. The parameters of the various methods were tuned to achieve the best possible results. All the images (original, noisy and restored) have been made available on KAGGLE, so that the research community can use them to compare their results with the output of the filters we have chosen for comparisons. You are right that the description of all the parameters would be advantageous, however a full list of their values, applied for each image of the dataset and noise intensity would be very lengthy and many huge tables would be required. Therefore we did not attempt to provide the detailed analysis of all used parameters of the various filtering techniques and their exact values. Instead we enabled the readers to download the final, optimal filtering result. If desired, the same filtering outputs can be obtained using the optimized combination of the filters' parameters described in the respective papers.

Reviewer 1:

> However, this part lacks a summary about the structure of this article, which will make the article easier to understand.

The brief summary has been added and the end of the introduction Section. Thank you for pointing out its absence, as it really improved the readability of the paper.

Reviewer 1:

> These algorithms are explained in detail, but it is better to do some horizontal comparisons between these methods.

If we have understood it correctly, the vertical comparison means evaluating the performance of a switching filter as a whole and the horizontal comparison means comparing detection and replacement algorithms separately. If our reasoning is correct, these kind of horizontal comparisons are already included in the paper. The separate comparison between detectors is presented in Tab. 1, where Accuracy and F1 scores are shown. For the impulse replacement part, the horizontal comparison is reflected by the evaluation performed using the Perfect Detector (PD), for which the impact of the detection performance is eliminated.

Reviewer 1:

> There is only one image being comparing in this part. More comparisons on other images should be provided in supplementary materials.

Thank you for the remark. Additional 6 thoroughly selected examples have been added to the paper as electronic supplementary material. We made additional comments on the efficiency of the evaluated filters showing their advantages and weaknesses depending on the image structure type and noise contamination intensity. We hope that the additional examples and also the included aim-plots will enable the reader to to get an insight into the filtering properties of the methods taken for comparisons.

Reviewer 1:

> Additionally, some relevant feature learning based techniques related to image restoration or impulse noise detection should be considered or at least analyzed...

Thank you for focusing our attention on the filtering methods based on learned dictionaries. We have included a short description of this denoising framework in the Introduction.

Reviewer 2:

> It will be a better if discussion of various detectors and estimators is put in compact way in a table/figure. This makes the discussion more compelling.

Thank you for this observation. We summarized all descriptions of both detection and estimation algorithms in one compact Table. We hope that such a modification improves the readability of the paper.

Once again we thank for the commitment to making our work more valuable. We can only hope that the provided revision will be considered as an improvement of the submitted paper.

Sincerely,
Lukasz Malinski
Krystian Radlak
Bogdan Smolka
